# An ecological study of geographic variation and factors associated with cesarean section rates in South Korea

**DOI:** 10.1186/s12884-019-2300-0

**Published:** 2019-05-09

**Authors:** Agnus M. Kim, Jong Heon Park, Sungchan Kang, Tae Ho Yoon, Yoon Kim

**Affiliations:** 1 Department of Health Policy and Management, Seoul National University College of Medicine, 103 Daehak-ro, Jongno-gu, Seoul, South Korea; 2 National Health Insurance Service, Wonju, Republic of Korea; 3 Graduate School of Public Health, Seoul National University, Seoul, Republic of Korea; 4 Department of Preventive & Occupational Medicine, School of Medicine, Pusan National University, Pusan, Republic of Korea; 5 Institute of Health Policy and Management, Medical Research Center, Seoul National University, Seoul, Republic of Korea

**Keywords:** Korea, Socioeconomic factors, Geographic variation, Cesarean section, Obstetric delivery, Deprivation, Poverty, Beds

## Abstract

**Background:**

Korea is in a condition where the impact of patient and supplier factors on cesarean section rates can be clearly described. The cesarean section rates in Korea are among the highest in the world while the number of obstetricians is decreasing sharply. This study aimed to investigate the geographic variation in cesarean section rates in Korea and its factors.

**Methods:**

The data were obtained from the National Health Insurance database in Korea in 2013. We calculated the age-standardized and crude cesarean section rates of 251 districts in Korea and variation statistics. A linear regression analysis was performed to determine factors for cesarean section rates.

**Results:**

The overall cesarean section rate in Korea was 364.6 cases per 1000 live births. The deprivation index score was strongly associated with the increase in the cesarean section rate while the density of hospital obstetricians and hospital beds showed a negative association. Average maternal age and total fertility rate showed a negative relationship with the cesarean section rate.

**Conclusions:**

Korea is suffering from a continuing decrease in obstetricians. Our study shows that this decline has more of an effect on mothers in the disadvantaged areas. Securing equal access to obstetric care among areas is necessary, and measures to encourage obstetricians and mothers not to opt for cesarean section are required.

**Electronic supplementary material:**

The online version of this article (10.1186/s12884-019-2300-0) contains supplementary material, which is available to authorized users.

## Background

The cesarean section is considered to be over-utilized worldwide. Cesarean section rates in a large majority of the globe are well above and even up to four times the tentative threshold of 10% over which no improvement of maternal and newborn mortality rates was found [1, 2]. The high cesarean section rate is the result of its extensive increase in recent decades across the globe, which was especially prominent in middle- and high-income countries. However, as medically non-indicated cesarean section can impose a significant burden on women’s health and medical expenditure in terms of its risk, cost, and complications weighed against its benefits [3–5], the rise in cesarean section rates raised public health concern and has been the focus of investigation in recent decades [6].

The rise in cesarean section rates is basically a result of the advances in medicine which made cesarean section a safe and convenient option [3]. However, women’s preference [7] and physicians’ preference [8–10], which were interwoven with the socio-cultural and practice environments, have also been found to be main drivers of the use of cesarean section. These characteristics were investigated at the individual and group level as factors for the high rate of cesarean section. However, the large gap in cesarean section rates within and across countries suggests that there are regional factors which prompt the use of cesarean section. Investigating the regional variation of cesarean section rates will enable an examination of its use in terms of regional characteristics, such as supply of obstetricians and regional socioeconomic conditions, in that the use of cesarean section can be affected by the supply of obstetricians and their opinions and is never separate from access to care.

Korea is in a condition where the impact of patient and supplier factors on cesarean section rates can be clearly elucidated. First, cesarean section rates in Korea are among the highest in the world [11]. Following a sharp rise from 4% in 1980 to 40% in 2000 [12], the cesarean section rates in Korea have remained above 35% ever since. These high cesarean section rates strongly suggest the likelihood that cesarean births were affected by external factors, such as the supply of obstetricians and their practice pattern, which may not be medically justifiable and could have resulted in unnecessary cesarean sections. Second, influence of factors for cesarean section are changing in Korea. While cesarean section was a preferred choice for the more affluent decades ago, the impact of wealth and education on cesarean section rate has been reversed [12]. Investigating factors for geographic variation in cesarean section rates will show the changing influence of the socioeconomic background on the use of cesarean section. Third, the practice environment of obstetricians in Korea has undergone changes which are not favorable to the obstetricians who would run the risk of unintended consequences of vaginal delivery. Medical malpractice lawsuits in Korea have continued to rise, and those involving obstetricians account for the highest expenses [13]. The recent introduction of a no-fault compensation scheme for physicians imposed an additional burden on obstetricians [14]. In addition, the low fee for vaginal delivery in Korea, which is about one fifth to one third of other developed countries [15, 16], also discourages obstetricians from opting for vaginal delivery [17]. As a result, the number of obstetric clinics, hospitals and new obstetricians in Korea continues to decrease at a sharp rate which is among the highest of all specialties. The number of health care facilities which perform child delivery decreased by half between 2004 and 2011, and the lack of obstetricians in rural areas became a serious social problem in Korea [18].

Examining geographic variation in cesarean section rates in Korea would indicate how these factors affected the use of cesarean section and help to identify the factors that maintain the high cesarean section rates in Korea. Therefore, this study will elucidate the factors of over-utilization of cesarean section which can adversely affect healthcare expenditure and women’s health. This study aimed to investigate geographic variation in cesarean section rates in Korea and its factors.

## Materials and methods

### Geographic variation in cesarean section rates

The data were obtained from the National Health Insurance (NHI) database in Korea in 2013. The NHI database contains health insurance claims data of the entire population of Korea, which are linked to information on patient demographics and health care facilities [19]. We collected the cases of cesarean section according to the procedure codes recorded in claims data. The number of live births was obtained from vital statistics of Statistics Korea in 2013.

Cesarean section rates were first calculated as the number of cesarean sections per 1000 live births and were standardized according to the ages of the mothers, which were divided into seven groups of 19 and under, 20–24, 25–29, 30–34, 35–39, 40–44, and 45 and above. The rates were calculated based on 251 districts in Korea. We performed the analysis with the standardized and crude rates respectively. To measure the regional variation in cesarean section rates, we calculated the ratio of the 90th percentile to the 10th percentile of the distribution of cesarean section rates (P90/P10), the coefficient of variation (CV), and the systematic component of variation (SCV). The SCV is a measure to estimate the true part of variation due to variation across areas by removing the random part of variation due to within-region variation [19–21]. As suggested by Mcpherson et al, the SCV was multiplied by 100 [19, 20]. 

### Factors for geographic variation in cesarean section rates

In order to investigate the factors affecting cesarean section rates, we performed a linear regression with the standardized and crude cesarean section rates as dependent variables. The independent variables are as follows: First, regarding socioeconomic circumstances, we used deprivation index which indicates the degree of socioeconomic deprivation in a region [22, 23]. The deprivation index was constructed with a total of nine items: proportion of population aged 65 and above, proportion of households without a car, proportion of households not living in apartments, proportion of households living below the minimum housing standard, proportion of population aged 35–64 without high school diplomas, proportion of households with household heads aged 15–64 employed in manual labor, proportion of people who were divorced, separated, or bereaved among those aged 15 or over, proportion of female headed households, and proportion of one person households [24, 25]. The data were obtained from the Population Census 2010 of Korea. The scores were standardized by a Z-score, and the weighted values were summated. Second, the average maternal age (average age of those who gave birth to children in 2013) was included. As the maternal age was only available at the 5 year interval, we applied the middle age of each age group to calculate average maternal age. Third, total fertility rate was included considering the possible relationship it can have with average maternal age and cesarean section rate. The data were provided by the Statistics Korea. Fourth, to assess how the density of obstetricians affects the cesarean section rate, we used the number of obstetricians per 100,000 population. As only a fourth of obstetrician clinics perform child delivery [26, 27], we used the number of obstetricians working in hospitals. Fifth, the number of hospital beds per 1000 was included to examine the impact of hospital bed density on cesarean section rates. The number of hospital beds was calculated excluding long term care hospitals, psychiatric hospitals, and rehabilitation hospitals. The data for the number of obstetricians and hospital beds were acquired from data issued by the Ministry of Health and Welfare [28].

Considering that the service area of hospital care is wider than districts [19], we applied two area levels to independent variables. Regarding socio-demographic conditions, the variables were calculated according to the district-level as in the cesarean section rate, and in the case of density of obstetricians and hospital beds, we applied the 56 hospital service areas (HSA). The HSA were organized to meet three criteria: 1. Localization index which indicates the proportion of health care use by the residents in an area [29]: 40% and above, 2. Population size: 150,000 and above, 3. Maximum transportation time within a region by car: 60 min and under. The HSA were made using the acute hospitalizations in Korea which occurred during 2011 and 2015 [30]. The model took the form:

Cesarean section rate_ij_ = β_0_ + β_1_ deprivation index score_i_ + β_2_ average maternal age_i_ + β_3_ total fertility rate_i_ + β_4_ number of obstetrician per 100,000_j_ + β_5_ number of hospital beds per 1000_j_ (_i_ = district, _j_ = hospital service area).

All the analyses were conducted using SAS, version 9.3 (SAS Institute, Inc., Cary, NC, USA) and SPSS 23 (IBM Corporation, Armonk, NY, USA).

## Results

**Fig. 1:**
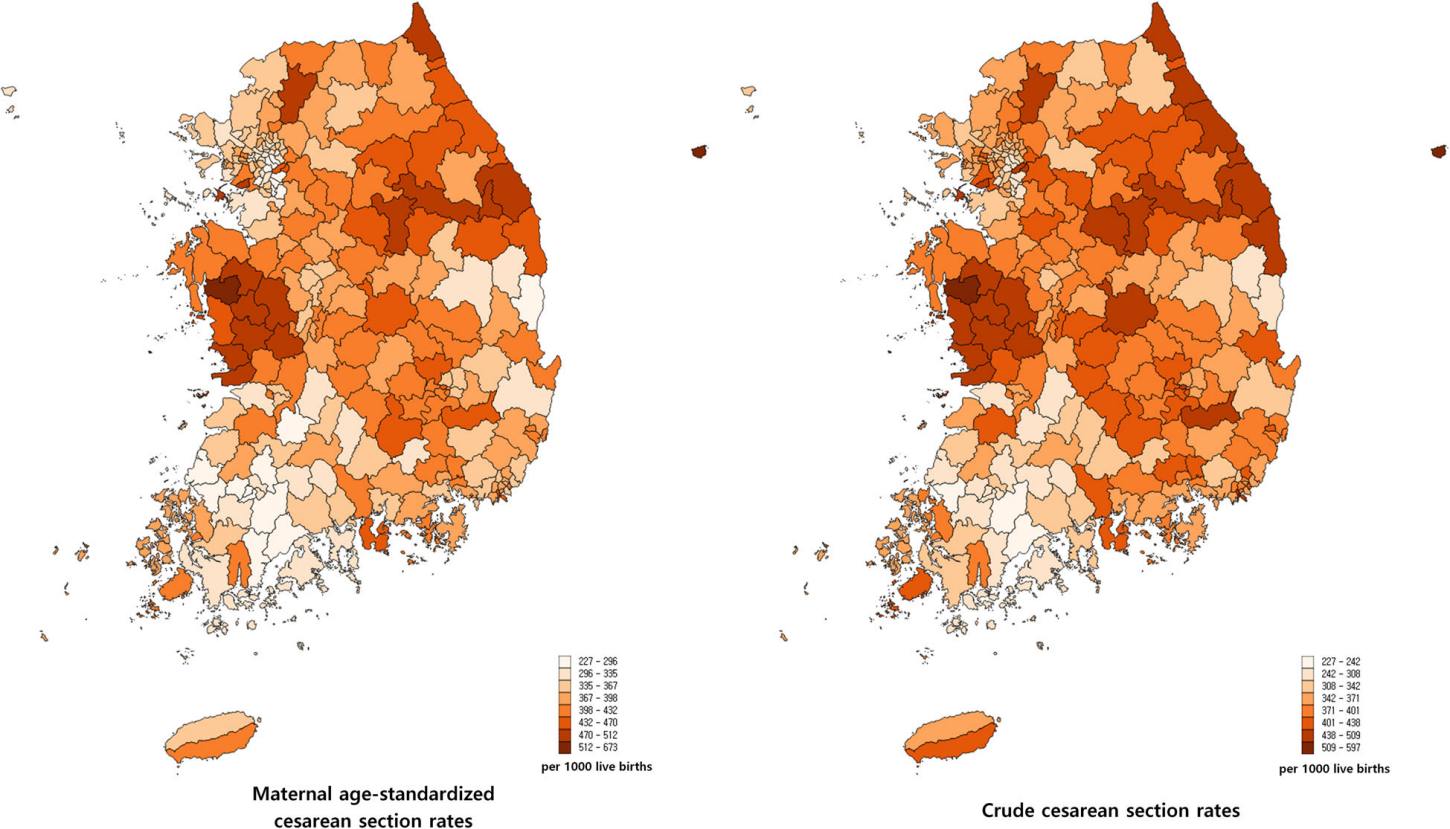
Map of cesarean section rates per 1000 live births in Korea in 2013. The figures were obtained using the data provided by the National Health Insurance Service in Korea.

There were a total of 159,934 cesarean sections in Korea in 2013 and the number of live births was 436,192. The national cesarean section rate was 364.6 per 1000 births. Figure 1 presents a map of the cesarean section rates at the district level. The crude cesarean rate ranged from 227.3 (Boseong-Gun) to 596.6 (Hongseong-Gun), and the standardized rate from 227.5 (Boseong-gun) to 680.3 (Ulleung-Gun). The rates tended to be higher in the South Chungcheong and Gangwon regions and lower in the South Jeonla and Seoul Metropolitan regions. The variation statistics of cesarean section rates are presented in Table 1.

**Table 1:** Variation statistics of cesarean section rates

	Standardized CS rate	Crude CS rate
Mean	380.6	374.0
Standard deviation	60.0	54.7
Max	227.5	227.3
Min	680.3	596.6
Max/Min	3.0	2.6
P90/P10	1.5	1.5
CV	0.2	0.1
SCV*	2.3	

*The SCV was multiplied by 100. *CS* cesarean section, Standardized CS rate, CS rate standardized to maternal age, *CV* coefficient of variation, *SCV* systematic component of variation

**Fig. 2:**
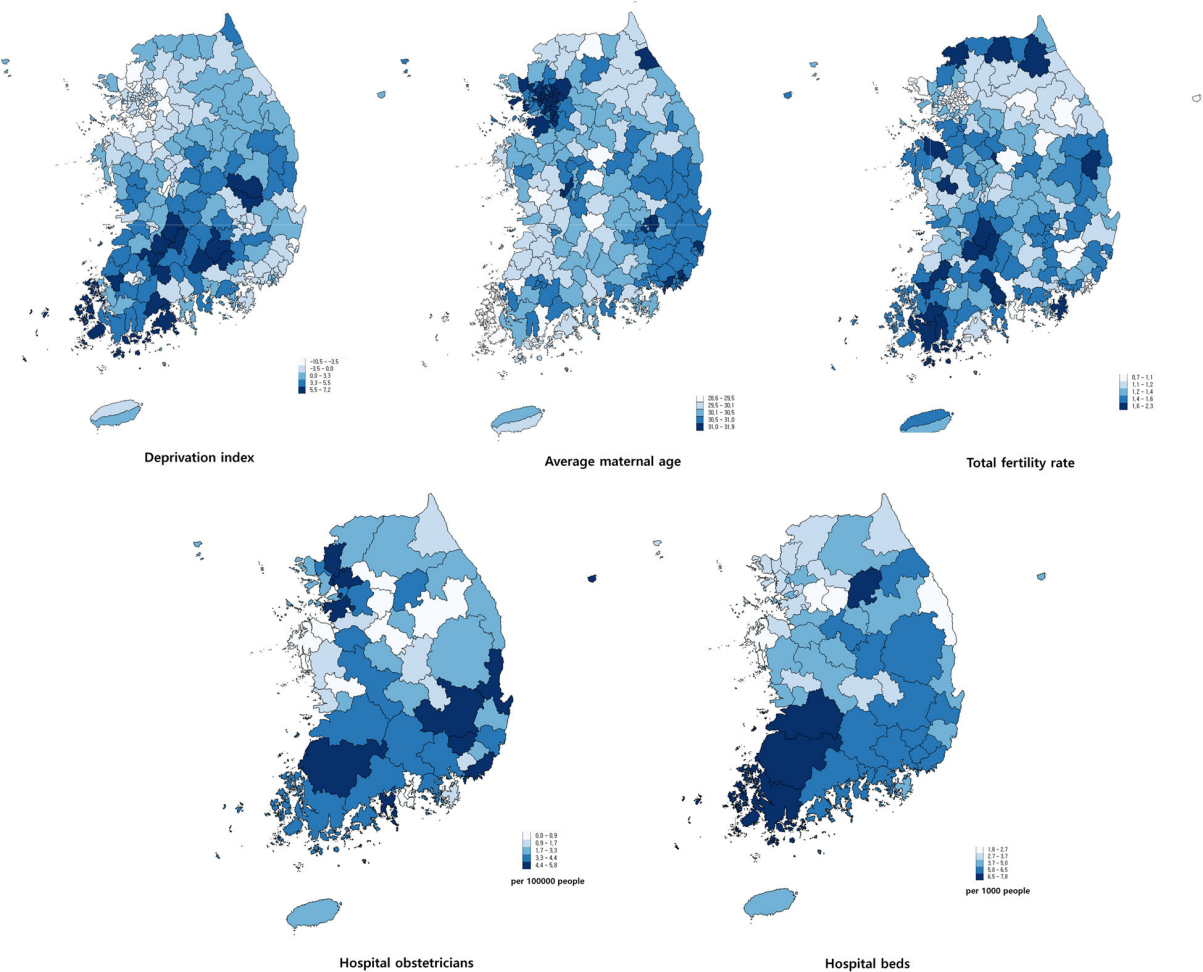
Map of explanatory variables in 251 districts (hospital obstetricians and hospital beds are based on the 56 hospital service areas). The figures were obtained using the data for the year 2013, except for the deprivation index which was based on the 2010 census

The characteristics of independent variables and their regional distribution are available in Fig. 2 and Additional file 1: Table S1. According to the regression analysis (Table 2), the deprivation index score was positively associated with the cesarean section rate, and the proportion of people aged 65 and over had a negative association. In the case of standardized cesarean section rate, a one unit increase in deprivation index score was associated with a 3.9 unit increase in the cesarean section rate. Considering the distribution of the deprivation index scores (Additional file 2: Table S2), the increase in the deprivation index score from the lowest to the mean means a 41.0 unit increase in the cesarean section rate, which is about 10% of the national cesarean rate. Average maternal age had a negative association with the standardized rate. A one unit increase in average maternal age was associated with a 26.1 unit decrease in the cesarean section rate. This indicates that its increase from the lowest to the mean (Additional file 2: Table S2) is related to a 52.2 unit decrease in the cesarean section rate. Total fertility rate showed a negative association with the cesarean section rate, and the increase in total fertility rate from the lowest to the mean is related to a 36.8 unit decrease in the cesarean section rate. Numbers of hospital obstetricians and hospital beds had a negative association with cesarean section rates. The increase in obstetricians and the increase in hospital beds from the lowest to the mean can be associated with 40.3 and 33.7 unit decreases in the cesarean section rate respectively.

**Table 2:** Regression analysis of cesarean section rates

Variables	Standardized CS rate		Crude CS rate	
	Coefficient	*P- value*	Coefficient	*P- value*
Baseline (intercept)	1347.609	<.001	1036.821	<.001
District-level				
Deprivation index	3.896	<.001	2.981	.014
Average maternal age	−26.092	.002	−15.748	.044
Total fertility rate	−61.364	<.001	−75.689	<.001
Hospital service area-level				
No. of hospital obstetricians per 100,000	−10.611	<.001	−9.967	<.001
No. of hospital beds per 1,000	−10.146	<.001	−9.376	<.001
R square	.314		.285	

*CS* cesarean section

## Discussion

This study investigated geographic variation in cesarean section rates in Korea and its factors using the 2013 National Health Insurance Database in Korea. The overall cesarean section rate in Korea was 364.6 cases per 1000 live births. Given the range of value of each variable and the size of the coefficient value, the deprivation index score showed a strong positive association with the cesarean section rate, while the hospital obstetrician density and hospital bed density showed negative associations. Average maternal age and total fertility rate showed negative relationships with the cesarean section rate.

Korea’s cesarean section rate was high compared with other countries as already shown in the other source [31]. Often, the increasing age of mothers is cited as a reason for this. However, despite the marked aging tendency of mothers in Korea, the age distribution of Korean mothers cannot be considered old when compared with other developed countries [32, 33], and the cesarean section rates were even higher in the 2000s when the mothers were relatively younger. Several factors could have contributed to the high cesarean section rates. From the mothers’ side, the convenience which cesarean section can provide could have been highly regarded [34], especially among working women whose numbers have been on a continuous rise [35]. For mothers to reconsider the cesarean, it is too accessible in terms of both expense and service supply. Additionally, the widespread practice of fixing the date of the birth in Korea for the purpose of giving the child a fortune could have precipitated the choice of cesarean section. Most of all, obstetricians in Korea are in a position that gives them little motivation to dissuade patients from cesarean section. Due to the increasingly high risk of medical litigation and low fee schedule for vaginal delivery, obstetricians have little reason for promoting vaginal delivery and risking the legal and financial problems [16]. Moreover, the decline in the number of obstetricians makes it more difficult for them to deal with untimely delivery, and this eventually leads to the preference for the mode of delivery for which scheduling is possible. The decreasing availability of obstetric facilities, especially in rural areas, could have also precipitated the use of cesarean section by reducing the opportunities for prenatal care [36].

Concerning geographic variation, the highest cesarean section rate was about three times that of the lowest district. This suggests that the likelihood of having a cesarean section can differ widely according to where the mothers live. Our further analysis provides a more elaborate account of how the regional characteristics affect the cesarean rates.

The positive association between the deprivation index score and cesarean section rate indicates that the mothers in more disadvantaged areas are more likely to have a cesarean delivery. This can be discussed from the regional and individual point of view. First, the poor regional circumstances could have caused its residents to be more susceptible to perinatal problems by its direct impact on health and by poor access to care. This is relevant given the sharp decrease in obstetricians in remote and rural areas in Korea. Our results, which showed an inverse relationship between the number of hospital obstetricians and cesarean section rates, also support this. In addition, as the areas with high deprivation scores have relatively fewer obstetricians, it would be difficult for suppliers to engage in deliveries during the night. As a result, they are more likely to encourage pregnant women to have a cesarean section.

Second, considering that the deprivation index is an aggregate index of individual socioeconomic conditions, the relationship between the deprivation index score and cesarean section rate can largely be explained as the relationship between the socioeconomic conditions and the likelihood of getting a cesarean section. In the past, the higher level of education and income was related to a higher likelihood to receive a cesarean section [37], and this tendency is still observed in less developed countries [38–40]. However, in recent studies in the US and other European countries, a negative relationship between the socioeconomic conditions and the use of cesarean section has been reported [41–43]. This change in the influence of socioeconomic conditions on the use of cesarean section was clearly observed in Korea over the past decades [12, 44]. Our results show that now in Korea, the cesarean section is being more frequently performed among those in the regions with less affluent conditions. In the past, the cesarean delivery was a medical procedure which seemed more sophisticated and was expensive; therefore, it was an available option for only those who could afford it [44]. But the cost of cesarean section declined, it became common, and greater recognition of its risks with increasing preference for the less artificial way of delivery could have reduced the preference for cesarean sections [15]. However, the reversal of the relationship between income and cesarean section rates is also likely to be related to the regional supplier factors.

The negative relationship between the number of hospital obstetricians and cesarean section rates can be one explanation. This shows that mothers in the areas with less hospital obstetricians are more likely to have cesarean sections. Considering that one quarter of all districts were left without health care facilities for child delivery for years [45], we can suppose that the lack of obstetric facilities could have negatively affected the access both to pre- and perinatal care, which led to higher cesarean section rates.

The number of hospital beds had a negative association with the cesarean section rates. This suggests that the regions with more hospitals offer favorable conditions for vaginal delivery and that the impact of hospital beds still remains even after allowing for the influence of the number of obstetricians. In viewing the oversupply of hospital beds in Korea [46], our results indicate that the oversupply is not universal among regions. Some regions may not benefit from the increase in hospital beds but rather suffer from the consequence of the lack of them.

The average maternal age had a negative association with cesarean section rate, and this relationship was distinctive in the standardized rate. This phenomenon is suggestive of possibilities: First, the overall cesarean section rate is lower in the regions with higher average maternal age, and, second, the cesarean section rate among the elderly mothers is lower in the regions with higher average maternal age. The cesarean section is more frequently performed among the aged mothers. The mothers in the urban and affluent areas are less likely to receive a cesarean section, and the average maternal ages in such regions tend to be higher than those in rural or deprived areas, which leads to a negative relationship between average maternal age and cesarean section rate. In addition, when comparing elderly mother groups, the urban and affluent areas have lower cesarean section rates. That is why the decreasing effect of average maternal age on the cesarean section rate is more distinctive in the standardized rates.

The inverse relationship between the total fertility rate and cesarean section rate shows that the scarcer the child delivery is, the more likely the cesarean section happens. Considering that this impact exists after the adjusting factors such as the average maternal age, density of hospital obstetricians, and hospital beds, we can suppose that the rarity of birth delivery in a region has its own impact of bringing down the proportion of natural birth. It is highly probable that a region where there are fewer child deliveries is not an attractive place for an obstetric practice. This relationship between the fertility rate and obstetrician supply needs to be further scrutinized as this can lead to a vicious cycle.

There are some considerations that should be taken into account when interpreting the results of this study. First, the unit of analysis in this study is a region, and the independent variables concerning mothers’ characteristics are aggregate variables. Therefore, the impact of those aggregate variables should be differentiated from their individual counterparts. The inverse relationship between the average maternal age and cesarean section rates is an example which shows that the influence of variables of regional unit and an individual can differ. As most of the studies investigating the factors for cesarean sections were performed with an individual as a unit of analysis, such differences should be taken into consideration when comparing our results with prior studies. Second, in July 2013, the diagnosis-related group (DRG) based payment system was introduced to several procedures including cesarean section. Although the cesarean section rate in Korea has been on the rise despite the application of the DRG based payment [47], the new payment system could have affected the behaviors of the health care providers, which this study could not have captured. Third, our results showed the distinctive geographic pattern of the cesarean section rates. Though we explained such differences with various variables, there is more that can explain the phenomenon. For example, the different practice patterns among regions could have resulted from the differences in training among regional training hospitals. This training factor is very important considering that a large number of obstetricians in a region are likely to have been trained in hospitals in that region. Therefore, the training factor should be investigated in future studies. Lastly, as the cesarean section rate in Korea continued to rise after 2013 [48], our study needs to be updated with the recent data, and longitudinal studies would make a more accurate investigation of factors for cesarean section possible. However, as a regional variation study, this study has found two important factors of cesarean sections: regional socioeconomic conditions and obstetrician density, both of which had a negative relationship with cesarean section rates. While reflecting the specific condition in Korea where a shortage of obstetricians became a serious issue, our study also shows the differing status of cesarean section as a medium of child delivery.

## Conclusions

Cesarean section rate in Korea is among the highest in the world, and there is a three-fold variation in the cesarean section rate in Korea. Through our analysis of geographic variation based on 251 districts and 56 hospital service areas, we found that socioeconomically disadvantageous conditions, lower density of obstetricians, and lower density of hospital beds were associated with higher cesarean section rates. Korea is suffering from the continuing decrease in obstetricians, with a quarter of all districts having no facilities for child delivery. Our study shows that this decline more heavily affects the mothers in the disadvantaged areas. Securing equal access to obstetric care among areas will be the first step toward decreasing the unnecessary cesarean sections. This should be followed by measures to encourage obstetricians and mothers not to opt for cesarean sections: Legal and financial protection for obstetricians and recognition of the risks of medically unjustified cesarean sections for mothers.

## Additional files

Additional file 1: **Table S1.** Characteristics of the independent variables. (DOCX 16 kb)

Additional file 2: **Table S2.** Correlation matrix of dependent and independent variables. (DOCX 15 kb)
