# Supplementary material for: An ecological study of geographic variation and factors associated with cesarean section rates in South Korea
Source: BMC Pregnancy Childbirth. 2019 May 9;19:162. doi: 10.1186/s12884-019-2300-0 (PMC6506939; doi:10.1186/s12884-019-2300-0)
Supplement: Supplementary file 1 — Table S1. Characteristics of the independent variables. (DOCX 16 kb) [file 12884_2019_2300_MOESM1_ESM.docx]

Additional file 1: Table S1. Characteristics of the independent variables

| Variables | Mean | Minimum | Maximum | Standard deviation |
| --- | --- | --- | --- | --- |
| District-level |  |  |  |  |
| Deprivation index | 0.0 | -10.5 | 7.2 | 3.6 |
| Average maternal age | 30.6 | 28.6 | 31.9 | 0.6 |
| Total fertility rate | 1.3 | 0.7 | 2.3 | 0.3 |
| Hospital service area-level |  |  |  |  |
| No. of hospital obstetricians per 100,000 | 3.8 | 0.0 | 5.8 | 1.5 |
| No. of hospital beds per 1,000 | 5.1 | 1.8 | 8.1 | 1.6 |
