# Supplementary material for: An ecological study of geographic variation and factors associated with cesarean section rates in South Korea
Source: BMC Pregnancy Childbirth. 2019 May 9;19:162. doi: 10.1186/s12884-019-2300-0 (PMC6506939; doi:10.1186/s12884-019-2300-0)
Supplement: Supplementary file 2 — Table S2. Correlation matrix of dependent and independent variables. (DOCX 15 kb) [file 12884_2019_2300_MOESM2_ESM.docx]

|  | Std CSR | Crude CSR | Dep | Mo. Age | TFR | H. Obs | H. Beds |
| --- | --- | --- | --- | --- | --- | --- | --- |
| Std CSR | 1 |  |  |  |  |  |  |
| Crude CSR | .969^**^ | 1 |  |  |  |  |  |
| Dep | .240^**^ | .119 | 1 |  |  |  |  |
| Mo. Age | -.336^**^ | -.193^**^ | -.661^**^ | 1 |  |  |  |
| TFR | -.052 | -.187^**^ | .300^**^ | -.405^**^ | 1 |  |  |
| H. Obs | -.405^**^ | -.347^**^ | -.158^*^ | .402^**^ | -.301^**^ | 1 |  |
| H. Beds | -.227^**^ | -.293^**^ | .476^**^ | -.266^**^ | .196^**^ | .356^**^ | 1 |

Additional file 1: Table S2. Correlation matrix of dependent and independent variables

***P* < 0.01, *Std CSR* standardized cesarean section rate, *Dep* deprivation index score, *Mo. Age* average maternal age, *TFR* total fertility rate, *H. Obs* hospital obstetricians per 100,000 people, *H. Beds* hospital beds per 1,000 people
